# Correction to “Generalizability of Enzyme‐Based Isolation Approach for Extracellular Vesicles from Traditional Medicinal Plants”

**DOI:** 10.1002/jex2.70096

**Published:** 2025-10-30

Wang, Q., R. Jing, N. Cao, et al. 2025. “ Generalizability of Enzyme‐Based Isolation Approach for Extracellular Vesicles From Traditional Medicinal Plants.” *Journal of Extracellular Biology* 4, no. 10: e70090. https://doi.org/10.1002/jex2.70090.

In the originally published article, the incorrect grant number was given for the grant from the Tianjin Belt and Road Project. The correct grant number is given below.

Incorrect

18JCQNJC79400

Correct

24PTLYHZ00330

We apologize for this error.

